# Role of sentinel lymph node biopsy for cutaneous melanoma in elderly patients: preliminary results in a Latin-American population

**DOI:** 10.3332/ecancer.2021.1167

**Published:** 2021-01-13

**Authors:** Carola Featherston, Walter Sebastián Nardi, Florencia Rocio Tomé, Sergio Damian Quildrian

**Affiliations:** 1 Department of General Surgery, British Hospital of Buenos Aires, Perdriel 74, CABA, 1280AEB, Buenos Aires, Argentina; 2 Sarcoma and Melanoma Unit, Department of General Surgery, British Hospital of Buenos Aires, Perdriel 74, CABA, 1280AEB, Buenos Aires, Argentina

**Keywords:** melanoma, sentinel lymph node biopsy, elderly patients

## Abstract

**Introduction:**

In melanoma, lymph node status constitutes the most important prognostic factor among patients with locoregional disease. It has been postulated that elderly patients present less metastatic involvement in sentinel lymph node (SLN). Our objective was to analyse the results and evolution of patients ≥ 70 years-old with cutaneous melanoma in whom sentinel lymph node biopsy (SLNB) was carried out.

**Methods:**

Retrospective analysis of 194 patients with primary CM who had a SLNB between 2005 and 2020 was included. Demographic and tumour data, SLN status, recurrence rate, morbidity and evolution were evaluated. Patients were divided into two groups according to age: Group 1 (<70 years old) and Group 2 (≥70 years old).

**Results:**

One hundred and fifty patients were included in Group 1 and 44 patients in Group 2. Median Breslow thickness was 1.7 mm in Group 1 and of 2 mm in Group 2 (*p* = 0.015).Forty-seven patients had positive SLNB; 38 (25%) belonged to Group 1 and 9 (20.5%) to Group 2 (*p* = 0.55). Recurrence was found in 34 patients: 25 belonging to Group 1 and 9 corresponding to Group 2 (*p* = 0.65). Morbidity was of 4% in Group 1 and 9% in Group 2 (*p* = 0.23). With an average follow-up of 30.6 months, 5-year overall survival was of 87% in Group 1 and of 63% in Group 2 (*p* = 0.04).

**Conclusion:**

Advanced age was not associated with differences regarding positivity of SLN and recurrence but difference in overall survival was observed. According to our results and the low morbidity rate, we consider SLNB should not be omitted in such age group, since it improves staging and gives the possibility to evaluate adjuvant treatment.

## Introduction

Since its implementation in the 90s, sentinel lymph node biopsy (SLNB) for cutaneous melanoma (CM) has been widely accepted and even started to be used for the management of other cutaneous tumours [1]. Currently, SLNB is a method of great value in the staging of patients with clinically negative lymph nodes. The possibility of metastatic compromise of the sentinel lymph node (SLN) is directly related to certain characteristics of the primary tumour, such as Breslow index and presence of ulceration. It is well known that in patients with thin melanomas (Breslow < 1 mm), metastatic spread is seen in 5%–8%, in intermediate-thickness melanoma is seen in 10%–15% of the cases and in thick melanomas (Breslow > 4 mm) the chances are higher than 40% [2]. Conversely, status of the regional lymph nodes in patients with clinically localised CM is the most important prognostic factor, given that melanoma-specific survival decreases significantly in patients with positive-SLN versus negative-SLN (62.1% ± 4.8% versus 85.1% ± 1.5%, respectively) [2, 3].

Primary tumour characteristics and the incidence of metastatic compromise of the SLN have been widely described in literature and, in a way, these factors have guided the decision making of SLNB. However, relationship between age and SLN compromise tends to be less clear [4–6]. Moreover, in elderly patients, the presence of comorbidities has been traditionally a relative contraindication for SLNB, specially taking into account that the final results of the Multicenter Selective Lymphadenectomy Trial (MSLT-I) have not shown advantages in terms of survival with this procedure. But with the development of new adjuvant treatments for locoregional disease (Stage III) that improve the outcomes of these patients, SLNB has gain a bigger role in the management of CM, even expanding the scope of its initial indications [7, 8]. With the increasing incidence of melanoma and ageing of the population in the last decades, we consider important to evaluate the implications of SLNB in elderly patients [9].

The objective of our study was to analyse the results and evolution of elderly patients (≥70 years) with primary CM who had SLNB at our institution and compare them with results obtained in younger patients.

## Methods

Approval from the Institutional Review Board was obtained for this study.

We performed a retrospective analysis from a prospective maintained database. We identified all patients with confirmed histopathological diagnosis of primary CM who had a SLNB performed from January 2005 through May 2020. Demographic, pathologic and outcome data were previously evaluated on individual medical records. Patients were divided into two groups according to age: Group 1 (<70 years) and Group 2 (≥ 70 years). All patients underwent lymphatic mapping using a radioactive colloid injected peri-tumourally or around scar of a previous excisional biopsy within 24 hours of SLNB. Lymphoscintigraphy and marking of SLNs were performed in all patients. In all cases, SLNB was realised under general anaesthesia. During surgery, blue dye was injected intradermically at the peri-lessional region by the surgeon. Then an incision was made over the marked area and sentinel node identification was made through both the visualisation of blue lymphatic and a gamma-probe to detect radiolabelled lymph nodes. Meticulous pathologic examination was done in all SLN using H&E and immunohistochemistry. In the same surgical procedure, definitive treatment of primary lesion was done as appropriate.

### Statistical analysis

Groups were compared using Fisher exact test for qualitative variables and Mann–Whitney test for quantitative variables. Kaplan–Meier method and Log-Rank test were used for survival analysis. A *p*-value less than 0.05 was considered statistically significant.

## Results

A total of 194 patients with primary CM underwent SLNB over the study period; 150 patients belong to Group 1 and 44 to Group 2. The mean age in the <70 years group was 51 years and 53% (80 patients) were men. Among the elderly group (≥70 years.), the mean age was 75 years and there was a greater predominance of men (33 patients, 75%) (*p* = 0.014). Median Breslow thickness for patients of Group 1 was 1.7 mm and for patients in Group 2 was 2 mm (*p* = 0.02). Location of melanomas was similar between groups except that older patients had higher proportion of head and neck melanomas (11.3% versus 2% in Group 1; *p* = 0.015). In <70 years patients, the most prevalent site was trunk (55%). Demographic and tumour characteristics are summarised in Table 1. As previously described, definitive treatment was done in the same surgical procedure. Wide surgical resection was performed in 20 patients with primary tumour *in situ* and in 175 patients with excisional biopsy scar.

SLN was detected in 193 of 194 procedures corresponding to 99.5% of SLN detection (150 in Group 1 and 44 in Group 2). Overall, prevalence of positive-SLN was similar between older patients and <70 years patients. SLN was positive in 47 (24%) patients; there were 38 (25%) of positive-SLN in Group 1 and 9 (20.5%) in Group 2 (*p* = 0.79). We detected 0.5% of false negative results. Overall complication rate was 5.1% (six patients in Group 1 versus four patients in Group 2; *p* = 0.23). Seven patients had grade I–II complications and three patients had grade III–IV complications: two haematomas that were surgically drained at postoperative day 1 and one patient who had a pulmonary thromboembolism requiring intensive care unit monitoring. There was no mortality associated to the procedure.

**Figure 1.**
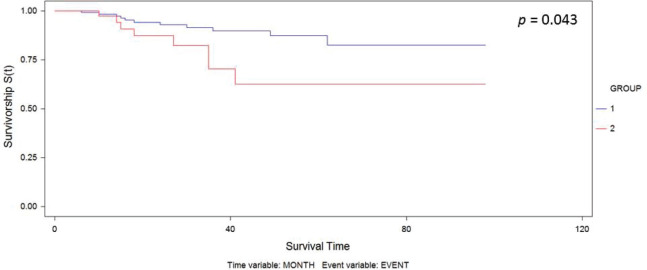
Kaplan–Meier curve for overall survival by group.

The median follow-up was 30.6 months. We observed recurrence in 34 patients (including local/in transit, regional or systemic recurrence); 25 patients (16.5%) belonged to Group 1 versus 9 patients (20.5%) to Group 2 (*p* = 0.65). The type of recurrence by group is shown in Table 2. Five-year overall survival was 87% and 63% in Group 1 and Group 2, respectively (*p* = 0.043) (Figure 1).

## Discussion

Information on CM in Latin America is scarce. A review of the published literature from six Latin American and Caribbean countries showed significant lack of information and differences in data collection, health system organisation and ethnicity throughout the region. The authors found that there were few studies reporting incidence and mortality rates to draw a reliable conclusion. However, it seems that the incidence has increased in the last years in line with world data. The geographic diversity of the region, the presence of native inhabitants and the European and African descendants conform a heterogeneous feature that sometimes complicates obtaining solid conclusions [10, 11]. The age-standardised incidence rates of CM per 100,000 people was established nearly 3–4.5 with a median age of 60 years, while mortality incidence was found to be 0.6–1.2/100,000 [12, 13]. Likewise, little information is founded about the relationship between age and prognosis in CM in this region.

The wide utilisation of SLNB in patients with CM has shown clear differences between age groups [4–6]. Elderly patients have a decreased SLN positivity rate, although they have poorer overall survival than younger patients. This observation is not linked to clinical and pathological characteristics of the primary tumour [14]. This lead some authors to suggest that melanoma in older patients spreads predominantly by haematogenous routes rather than lymphatic ones [9]. Understanding of the mechanisms associated to heterogeneity by age group in melanoma is limited. Some authors support age-related changes in immunologic functions and a decreased lymphatic flow to lymphatic nodes. Others propose that some variations in the host biology may influence in the progression of the disease, such as sarcopenia, which is more prevalent among older patients [15]. However, results of SLNB in CM and the impact of this procedure in the older population are usually difficult to interpret, in part because these patients are sometimes underrepresented or excluded from studies [16].

Several authors have postulated that, although SLNB is an outpatient or short-stay procedure, it is usually underused in elderly patients. This behaviour may be based both on the associated comorbidities of this age group, decreasing the possibilities of performing procedures that in many cases require general anaesthesia, and on the limited scientific evidence of their usefulness in these patients [16].

Numerous studies describe a decrease in the incidence of SLN positivity and a lower survival in elderly patients [14, 17]. In our study, advanced age was not associated with significant differences in SLN positivity or disease recurrence but overall survival was poorer according to published data. However, other authors such as Rees *et al* [18] could not confirm that increasing age was a significant independent factor of survival, suggesting that these variations could be due to increased comorbidities in older patients and not to age itself. Sabel *et al* [19] found statistically significant SLNB positivity in elderly patients with melanoma of 1 mm Breslow depth or thicker, so they recommend performing it in selected patients, since it provides prognostic information associated with better results. In the study published by Grotz *et al* [9] in which 358 patients older than 65 years were included, the detection of the SLN was 98%, with high sensitivity and a low rate of false negative results, which concludes that age alone should not be a contraindication for SLNB. Koskivuo *et al* [16] studied 423 patients older than 70 years and obtained similar results, concluding that SLNB is an accurate technique to detect nodal metastases in older patients and is the most important prognostic factor.

Recent prospective randomised control trials have evaluated the utility of adjuvant treatments in patients with lymph node metastases (Stage III) providing improvements in survival. This finding supports the importance of better staging in patients with CM [7, 8, 20]. Advances in adjuvant therapy highlight the importance of correct staging in patients with clinically localised CM who may have subclinical lymph node disease and who could benefit from these treatments. This new evidence could lead to reconsidering the usefulness and indications for SLNB and not only suggest it in patients with CM of intermediate thickness (1–4 mm) or thin melanomas (<1 mm) with poor prognosis factors. Knowing that patients with thick melanomas (>4 mm) and clinically negative nodes may have an even worse prognosis than those with thinner melanomas and little sentinel node tumour burden, better staging may benefit them through adjuvant treatment. The same criteria can be applied for patients with advanced age, since before having adjuvant therapies for stage III patients, it might not be justified to submit them to a SLNB, which is known to have no impact per se on disease-specific survival as demonstrated in MSLT-I. But now, given the new evidence, the procedure takes on additional importance in older patients [2]. The SLNB provides important prognostic and staging data with minimal morbidity and allows identification of patients with microscopically positive nodes who would benefit from adjuvant treatments [2, 21].

Based on the results of MSLT-I trial the clinical guidelines recommended complete lymph node dissection in patients with SLN metastases. This was another point of controversy because MSLT-I did not show a survival benefit performing SLNB and an immediate CLND was recommended in SLN-positive patients. This behaviour forced to a second procedure, increasing the risks, and was another argument to avoid SLNB in elderly patients. This indication changed dramatically after two prospective randomised clinical trials. MSLT-II and De-COG SLT trials did not demonstrate survival benefit with immediate CLND after a SLN-positive versus strict nodal observation.

These trials showed that immediate CLND increases regional disease control and provided prognostic information but this is not associated with increased melanoma-specific survival [22, 23]. Taking into account the positive results of adjuvant clinical trials in Stage III melanoma patients and the lack of evidence of immediate CLND, it is necessary to rethink the management in advanced age patients with CM. SLNB would improve staging, allowing the patient to receive an adjuvant treatment, without the requirement of immediate CLND and thus improving survival and decreasing the final morbidity.

Management of regional lymph nodes in patients with melanoma has changed drastically over the last years, from a procedure destined to therapeutic purposes to one in which prognostic information is nowadays of primordial importance. The role of SLNB is decisive in patients with clinical negative nodes, and complete dissection is still a standard of care in those patients with clinically positive regional disease. Advances in adjuvant treatments make prognostic information critical in order to correctly select patients who may benefit from adjuvant therapies [24].

## Conclusion

While ≥70-year-old patients had tumours with greater Breslow thickness in our study, advanced age was not associated with differences regarding SLN positivity or disease recurrence rate but it is important to note that overall survival was worse compared with younger patients. Taking into account our results and the low morbidity rate associated with the procedure, we consider SLNB should not be omitted in this age group.

## List of abbreviations

SLNB, sentinel lymph node biopsy; CM, cutaneous melanoma; SLN, sentinel lymph node; MSLT-I, Multicenter Selective Lymphadenectomy Trial; CLND, completion lymph node dissection.

## Conflicts of interest

None.

## Funding

No funding was received for this article.

## Authors’ contributions

Study concept and design: SQ, WN. Acquisition of data: CF, FT, WN. Drafting of the manuscript: SQ, WN, CF. Critical revision of the manuscript for important intellectual content: SQ. Final revision and final approval for publication: SQ. All authors read and approved the final manuscript.

## Figures and Tables

**Table 1.** Patient and melanoma characteristics by age group.

Primary tumour characteristics	Group 1^a^ n = 150	Group 2^b^ n = 44	Total n = 194
*Male*	80 (53%)	33 (75%)	113 (58%)
*Female*	70 (47%)	11 (25%)	82 (42%)
*Median age*	51 (9–69)	75 (70–84)	
***Location*** *Head and neck* *Trunk* *Extremity*	3 (2%) 83 (55%) 64 (43%)	5 (11.3%) 23 (52.3%) 16 (36.4%)	8 (4%) 106 (54.5%) 81 (41.5%)
***Median Breslow index*** ***T stage*** *T1* *T2* *T3* *T4*	1.7 (0.1–9) 38 (25%) 58 (39%) 40 (26.5%) 14 (9.5%)	2 (0.8–15) 10 (22.7%) 12 (27.3%) 13 (29.5%) 9 (20.5%)	- 48 (24.5%) 71 (36.5%) 53 (27%) 23 (12%)
***Ulceration***	38 (25%)	16 (36.5%)	54 (27.6%)
***mitotic rate*** *<1 mm2* *>1 mm2* *No data*	47 (31%) 74 (49%) 30 (20%)	12 (27.3%) 24 (54.5%) 8 (18.2%)	59 (30.2%) 98 (50.3%) 38 (19.5%)

a <70 years

b ≥70 years

**Table 2.** Recurrence type by age group.

	Group 1^a^	Group 2^b^	Total
***Local/regional***	7 (4.6%)	1 (2.3%)	8 (4.1%)
***Distant***	10 (6.6%)	7 (15.9%)	17 (8.7%)
***Both***	8 (5.3%)	1 (2.3%)	9 (4.6%)
***Total***	25 (16.5%)	9 (20.5%)	34 (17.4%)

a <70 years

b ≥70 years
